# Supplementary material for: Effects of Pulsed Electric Field Pretreatment on Black Tea Processing and Its Impact on Cold-Brewed Tea
Source: Foods. 2024 Jan 3;13(1):164. doi: 10.3390/foods13010164 (PMC10779252; doi:10.3390/foods13010164)
Supplement: Supplementary file 1 [file foods-13-00164-s001.zip › foods-2768588-supplementary.pdf]

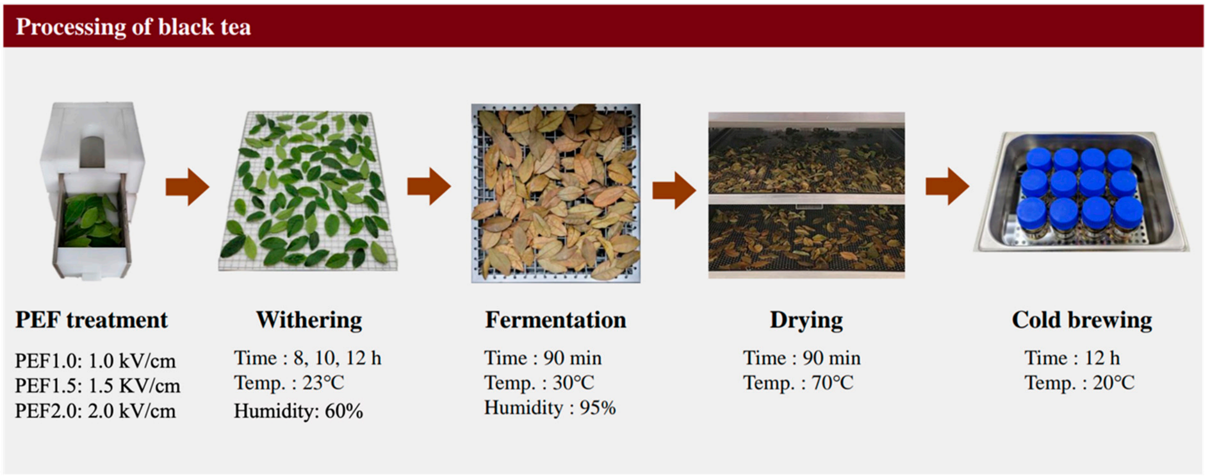

Figure S1. The process flow for the PEF treatment, withering, fermentation, drying and cold b rewing with detailed conditions.
